# Methylation status and long‐fragment cell‐free DNA are prognostic biomarkers for gastric cancer

**DOI:** 10.1002/cam4.3755

**Published:** 2021-02-28

**Authors:** Kazuhide Ko, Yoshikazu Kananazawa, Takeshi Yamada, Daisuke Kakinuma, Kunihiko Matsuno, Fumihiko Ando, Sho Kuriyama, Akihisa Matsuda, Hiroshi Yoshida

**Affiliations:** ^1^ Department of Gastrointestinal and Hepato‐Biliary‐Pancreatic Surgery Nippon Medical School Tokyo Japan

**Keywords:** biomarker, circulating cell‐free DNA, gastric cancer, LINE‐1, repeated DNA sequences

## Abstract

**Background:**

Circulating tumor DNA (ctDNA) detected before surgery disappears after complete surgical resection of the cancer. Residual ctDNA indicates minimal residual disease (MRD), which is a cause of recurrence. The presence of long‐fragment circulating cell‐free DNA (cfDNA) or methylated cfDNA also implies the presence of cancer. In this study, we evaluated the prognostic value of cfDNA methylation and long‐fragment cfDNA concentration in gastric cancer patients undergoing curative surgery

**Methods:**

Ninety‐nine gastric cancer patients were included. Peripheral blood samples were collected before and 1 month after surgery. In patients administered chemotherapy, samples were collected before starting chemotherapy. qPCR was performed to detect long‐ and short‐fragment LINE‐1. A plasma HELP (HpaII tiny fragment Enrichment by Ligation‐mediated PCR) assay to determine the concentration of HpaII small fragments was performed using ligation‐mediated PCR and HpaII was quantified as the HpaII:MspI ratio to detect methylation levels of cfDNA.

**Results:**

Overall survival (OS) of patients with low methylation levels before starting treatment was significantly worse than that of patients with high methylation levels (*P* = 0.006). In the 90 patients who underwent curative surgery, recurrence‐free survival (RFS) and OS of patients with low methylation levels before surgery were worse than those with high methylation levels (P=0.08 and *P* = 0.11, respectively). RFS and OS of patients with high concentrations of long‐fragment LINE‐1 after surgery were significantly worse than those with low concentrations of long‐fragment LINE‐1 (*P* = 0.009, *P* = 0.04).

**Conclusions:**

Pre‐surgical low methylation levels of LINE‐1 are a negative prognostic factor. Post‐surgical high concentrations of long‐fragment LINE‐1 indicate MRD and a high risk of recurrence.

## Graphical abstract

Presurgical hypomethylation of LINE‐1 in cfDNA is a negative prognostic factor and the methylation level decreases at the time of recurrence. High concentrations of long LINE‐1 after curative surgery indicate minimal residual disease and high risk of recurrence. Serial liquid biopsy to detect LINE‐1 methylation levels and long LINE‐1 concentration can yield useful information for determining the treatment strategy for patients with gastric cancer.

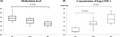

## 1 INTRODUCTION

Gastrectomy is the only potentially curative treatment for patients with advanced gastric cancer; however, patients who undergo curative surgery often develop recurrent disease. This indicates that localized disease includes potentially systemic disease. Therefore, presurgical diagnosis of potential systemic recurrence and postsurgical diagnosis of minimal residual disease (MRD)^1^, ^2^ can improve the outcome of curative gastrectomy. In patients recovering from complete surgical resection of cancer, no circulating tumor DNA (ctDNA) is detected after surgery due to its short half‐life. Thus, the presence of ctDNA after surgery theoretically indicates the presence of MRD.^3^ This is because tumor cells that circulate in the blood and micro‐metastatic deposits at distant sites can also release ctDNA.^4^

Circulating cell‐free DNA (cfDNA), derived from both normal and cancer cells, exists in the blood.^4^ In particular, cfDNA that is derived from tumors and that possesses tumor‐specific mutations is called ctDNA.^5^ We have already reported that we can use cfDNA and ctDNA for various purposes, including assessment of MRD in patients with colorectal cancer.^2^ ^,^ ^6^, ^7^, ^8^, ^9^, ^10^ ctDNA is also a useful biomarker for patients with gastric cancer.^11^ However, it is often not detected in these patients. According to the molecular subtype classification of gastric cancer, only the microsatellite unstable subtype shows hyper‐mutation, accounting for approximately 20% of cases; however, another three subtypes show hypo‐mutation.^12^ Thus, ctDNA is often not detected in patients with other subtypes; hence development of novel nucleic acid biomarkers other than ctDNA is required for patients with gastric cancer.

Long interspersed nuclear element‐1 (LINE‐1) constitutes approximately 17% of the human genome.^13^ Therefore, LINE‐1 cfDNA is present in the blood of most people. Alterations in the methylation status of LINE‐1 elements are a surrogate marker for global DNA methylation, which is one of the distinguishing features of various cancers. LINE‐1 methylation is associated with more aggressive progression of cancer.^14^ Many studies have reported that LINE‐1 methylation in cancer tissues is associated with cancer risk, progression, and poor prognosis.^15^ However, only a few studies have reported the prognostic value of LINE‐1 methylation status in cell‐free DNA.^16^, ^17^

In healthy individuals, the main source of cfDNA is apoptotic cells. Conversely, cfDNA derived from cancer cells originates from apoptotic and necrotic cells.^18^ Short‐fragment (<150 bp) cfDNA is derived from apoptotic or necrotic cells. In contrast, most long‐fragment (>150 bp) cfDNA is derived from necrotic cells, including necrotic cancer cells. The concentration and proportion of long‐fragment cfDNA is high in cancer patients.^19^ Thus, the existence of long‐fragment cfDNA indicates the presence of cancer cells, while that after curative surgery can indicate MRD and may be a risk factor for recurrence. We previously reported that the presence of LINE‐1 long‐fragment cfDNA (long LINE‐1) after surgery indicates MRD and is a risk factor for recurrence after liver metastasectomy from colorectal cancer.^2^

In this study, we evaluated the prognostic value of cfDNA methylation and the concentration of long‐fragment cfDNA pre‐ and postsurgery in gastric cancer patients undergoing curative surgery. The development of a novel prognostic biomarker for pre‐ or postsurgery could indicate patients who require neoadjuvant chemotherapy or adjuvant chemotherapy after surgery.

## 2 MATERIALS AND METHODS

### 2.1 Patients

This was a single‐center, observational study. A consecutive series of patients with stomach adenocarcinoma treated from October 2016 to March 2018 were included in this study. Inclusion criteria were: (i) diagnosis of adenocarcinoma of the stomach; (ii) patients who underwent elective surgery or chemotherapy (unresectable case); and (iii) age >20 years. Exclusion criteria were: (i) second malignancies; (ii) serious comorbidities such as chronic heart failure, chronic renal disease, or connective tissue disease; and (iii) clinical history of other organ cancer. We also included eight control patients with benign disease.

This study was carried out in accordance with the Declaration of Helsinki. The study protocol was approved by the ethics review committee of our institution (approval number: 28‐03‐738). Written informed consent was obtained from each patient.

### 2.2 Clinical follow‐up

Blood samples were collected from all patients to measure carcinoembryonic antigen (CEA) and CA19‐9 at every outpatient visit. Every patient visited the outpatient clinic 1 month after surgery and then every 3–6 months thereafter. Computed tomography imaging of the chest and abdomen was performed every 6 months. Examinations were performed immediately if CEA (>5 ng/ml) or CA19‐9 (>37.0 U/ml) concentration was increased. Every year after surgery, upper endoscopy was performed to detect local recurrence. Recurrence‐free survival (RFS) was defined as the time from the surgery to either recurrence or death from any cause.

### 2.3 Sample collection

Peripheral blood samples were collected before surgery and 1 month afterward. In patients who experienced recurrence, blood samples were collected at the time of recurrence. In patients given chemotherapy, samples were collected before starting chemotherapy. Samples were transferred to BD Vacutainer EDTA tubes (Becton Dickinson) and processed within 2 h. Plasma was separated by centrifuging the blood at 3000*g* for 10 min at 4°C and then stored at −80°C until DNA extraction, as previously reported.^6^, ^7^, ^8^

cfDNA was extracted from 1 ml of plasma using the Maxwell® RSC cfDNA Plasma Kit (Promega) according to blood and fluid protocols recommended by the manufacturer. cfDNA concentrations were measured using the Qubit quantification assay (Thermo Fisher Scientific).

### 2.4 Quantitative polymerase chain reaction (qPCR) of repeat DNA sequences

qPCR was performed to detect LINE‐1 using primers that amplify >97‐bp fragments (L‐97) and >169‐bp fragments (L‐169). L‐97 represents the short fragment of LINE‐1 in cfDNA (short LINE‐1) and the total amount of LINE‐1 gene fragments in cfDNA. L‐169 represents the long fragment of LINE‐1 in cfDNA (long LINE‐1).^20^, ^21^ The DNA integrity index was calculated as the ratio of L‐169 and L‐97 in each sample.^2^, ^20^, ^21^, ^22^ The primer set for L‐97 amplified the short DNA segment targeting the most abundant 5 ‘UTR of the LINE‐1 sequence, forward: 5′‐TGGCACATATACACCATGGAA‐3′, and reverse: 5′‐GAGAATGATGGTTCTCCAATTTC‐3′.^23^ We designed new L‐169 primers as follows: forward, 5′‐GACGGGTGATTTCTGCATTT‐3′ and reverse, 5′‐ TCACCCCTTTCTTGACTCG‐3′. A mixture containing primers and cfDNA with a total volume of 20 μl was used to perform qPCR. The amplification reaction was performed using the Applied Biosystems 7900 HT High‐Speed Real‐Time PCR System (Applied Biosystems) according to the manufacturer's protocols. All qPCR assays were analyzed in duplicate without knowing the identity of the samples. In our previous report,^2^ long LINE‐1 was defined as fragments >300 bp. However, the reproducibility of LINE‐1 >169 bp was higher than that of LINE‐1 >300 bp.

### 2.5 Measurement of plasma LINE‐1 methylation

A plasma HELP (HpaII tiny fragment Enrichment by Ligation‐mediated PCR) assay to determine the concentration of *HpaII* small fragments by ligation‐mediated PCR, using methylation restriction isoschizomer enzymes,^24^, ^25^ was performed and quantified as *HpaII*:*MspI* ratio to detect the methylation levels of cfDNA. A low ratio indicated hypomethylation.

### 2.6 Statistical analysis

All statistical analyses were performed using SPSS version 23.0 software (IBM Corp.). Comparisons between groups were analyzed using the chi‐squared (*χ* ^2^) test or Fisher's exact test for categorical variables. The Mann–Whitney *U* test or the Kruskal–Wallis test was used to analyze quantitative variables. A receiver operating characteristic (ROC) curve and the respective area under the ROC curve were calculated to distinguish patients with gastric cancer from patients with benign disease, and to distinguish patients who experienced recurrence from patients who did not. Differences in RFS and OS were examined using the log‐rank test and Cox regression analysis. Multivariate analysis of prognostic factors related to RFS was performed using a Cox proportional hazard analysis. In all analyses, a value of *p* < 0.05 was considered significant. We hypothesized that the 3‐year survival rate of the low‐risk group was 85% and that of the high‐risk group was 60%. Power analysis (alpha = 0.05, Power‐0.8) showed that 76 patients were needed. Thus, we planned to include 90 patients who underwent curative surgery.

## 3 RESULTS

### 3.1 Patients

Over the study duration, 128 patients with gastric cancer were treated, and 29 patients were excluded because of multi‐organ malignancy (*n* = 8), serious comorbidities (*n* = 6), endoscopic treatment (*n* = 3), and neoadjuvant chemotherapy (*n* = 12). Thus, 99 patients with gastric cancer and eight patients with benign disease (seven patients with duodenal ulcers and one patient with a gastric ulcer; five males and three females with a median age of 70 years) were included. Patient backgrounds are shown in Table 1. Curative surgery was performed on 90 patients (90.1%). Forty‐eight patients with stage IIB or III disease required adjuvant chemotherapy using S‐1. Of these, 12 patients did not receive adjuvant chemotherapy because of advanced age (>75 years, *n* = 6), patient refusal (*n* = 3), or comorbidities (*n* = 3). Eighteen of the 36 patients who received adjuvant chemotherapy completed adjuvant chemotherapy in 12 months.

**TABLE 1:** Patients’ background

Variable	Patients *N*	%	Variable	Patients *N*	%
Sex			*T*		
Male	80	80.1	1	34	34.3
Female	19	19.9	2	10	10.1
Age	72 (36–89)		3	27	27.3
Tumor location		4	28	28.3	
Upper	21	21.2	*N*		
Middle	44	44.4	0	50	50.5
Lower	34	34.3	1	21	21.2
Tumor size			2	12	12.1
<30 mm	32	27.8	3	16	16.2
≥30 mm	67	72.2	Venous invasion		
Pathological stage			Negative	31	31.3
I	36	36.4	Positive	68	68.7
II	27	27.3	Lymphatic invasion		
III	27	27.3	Negative	26	26.3
IV	9	9.1	Positive	73	73.7
			Histolopathology		
			Differentiated	50	50.5
			Undifferentiated	49	49.5

### 3.2 cfDNA concentration

cfDNA concentrations are shown in Table 2. The median cfDNA concentration in benign disease was 219 (range, 164–370) pg/μl and that of patients with gastric cancer was 205 (range, 28–1373) pg/μl, with no significant difference (*p* = 0.31). Tumor size (*p* = 0.04) was significantly associated with higher cfDNA concentrations. cfDNA concentrations tended to be higher in stage IV gastric cancer patients; however, there was no significant difference in clinical stage (*p* = 0.26).

**TABLE 2:** cfDNA, Short LINE‐1 and Long LINE‐1 concentration and cell‐free LINE1 methylation levels (HpaI/MspII ratio)

	cfDNA (pg/ml) Median (IQR)	*p*	Short LINE‐1 (copies/pg) Median (IQR)	*p*	Long LINE‐1 (copies/pg) Median (IQR)	*p*	Methylation level Median (IQR)	*p*
Sex		0.03		0.45		0.09		0.51
Male	230 (196)		1.20 (1.25)		0.35 (0.50)		4.11 (1.39)	
Female	169 (78)		1.58 (1.65)		0.29 (0.42)		3.81 (2.11)	
Benign disease	219 (139)	0.31	1.21 (1.84)	0.87	0.67 (0.57)	0.04		0.006
Gastric cancer	205 (194)		1.42 (1.34)		0.35 (0.45)		4.98 (1.37)	
Tumor location		0.37		0.06		0.40	3.91 (1.45)	0.17
Upper	208 (132)		1.71 (1.05)		0.35 (0.60)			
Middle	181 (211)		1.46 (1.61)		0.30 (0.45)			
Lower	233 (179)		1.43 (0.66)		0.35 0.39)		3.14 (2.09)	
Tumor size		0.04		0.11		0.29	3.98 (1.27)	0.91
<30 mm	169 (124)		1.66 (1.20)		0.35 (0.44)		4.27 (1.23)	
≥30 mm	226 (250)		1.43 (1.41)		0.31 (0.52)			
Pathological stage		0.26		0.49		0.96	3.98 (1.47)	
I	197 (185)		1.15 (1.44)		0.31 (0.44)		3.90 (1.51)	0.04
II	177 (176)		1.17 (1.20)		0.36 (0.58)			
III	219 (170)		1.50 (1.31)		0.29 (0.71)			
IV	404 (364)		1.42 (1.17)		0.35 (0.44)		3.79 (1.42)	
*T*		0.61		0.16		0.19		0.70
1	184 (183)		1.15 (1.19)		0.32 (0.47)		3.56 (1.52)	
2	228 (686)		0.65 (1.20)		0.25 (0.48)		3.88 (0.91)	
3	178 (151)		1.44 (1.63)		0.30 (0.45)		4.14 (1.80)	
4	208 (339)		1.46 (1.2)		0.36 (0.63)		3.92 (1.77)	
*N*		0.66		0.47		0.31		0.23
0	187 (185)		1.15 (1.44)		0.35 (0.46)		4.10 (1.66)	
1	226 (213)		1.43 (1.02)		0.29 (0.32)		3.46 (1.89)	
2	230 (275)		1.15 (1.44)		0.49 (0.84)		4.19 (0.99)	
3	217 (328)		1.53 (1.26)		0.38 (0.33)		3.95 (1.24)	
Venous invasion		0.07		0.03		0.25		0.73
Negative	172 (102)		1.13 (1.37)		0.23 (0.56)		4.08 (1.23)	
Positive	226 (211)		1.43 (1.22)		0.35 (0.37)		3.91 (1.57)	
Lymphatic invasion		0.41		0.04		0.60		0.69
Negative	181 (174)		0.57 (1.82)		0.25 (0.55)		3.49 (1.67)	
Positive	208 (197)		1.43 (1.16)		0.34 (0.37)		3.95 (1.48)	
Histopathology		0.23		0.83		0.90		0.61
Differentiated	221 (172)		1.18 (1.21)		0.29 0.53)		4.16 (1.47)	
Undifferentiated	169 (170)		1.40 (1.47)		0.35 (0.39)		3.86 (1.38)	

Abbreviations: cfDNA, Circulating cell‐free DNA; IQR, interquartile range.

### 3.3 LINE‐1 concentration

Short LINE‐1 (L‐97) and long LINE‐1 (L‐169) concentrations are shown Table 2. There was no difference between the short LINE‐1 concentration of patients with gastric cancer and that of patients with benign disease (*p* = 0.87). However, the long LINE‐1 concentration in patients with gastric cancer was significantly lower than that in patients with benign disease (*p* = 0.04). Short LINE‐1 concentration was higher in positive venous invasion (*p* = 0.03) and positive lymphatic invasion (*p* = 0.04); however, stage had no impact on the concentration of short LINE‐1 (*p* = 0.49). There was no correlation between long LINE‐1 concentration and clinicopathological factors.

### 3.4 LINE‐1 methylation level in cfDNA

Median Hpa II, Msp I and Hpa:Msp I ratio are 7.22 (IQR: 4.27–14.96), 1.80 (IQR: 0.84–3.67), and 3.90 (IQR: 3.23–4.65), respectively. LINE‐1 methylation levels (*HpaII*:*MspI* ratio) are shown in Table 2. LINE‐1 methylation levels in patients with gastric cancer were significantly lower than those in patients with benign disease (*p* = 0.006). In the ROC curve for distinguishing patients with gastric cancer from patients with benign disease, the area under the curve (AUC) was 0.81 (95% confidence interval [CI]: 0.71–0.92; *p* < 0.001). It is noteworthy that the methylation level of patients with stage I gastric cancer was significantly lower than that of patients with benign disease (*p* = 0.005). Furthermore, the median LINE‐1 methylation level in patients with stage IV disease was significantly lower compared with stage I–III patients (*p* = 0.04).

### 3.5 Prognostic value

**FIGURE 1:**
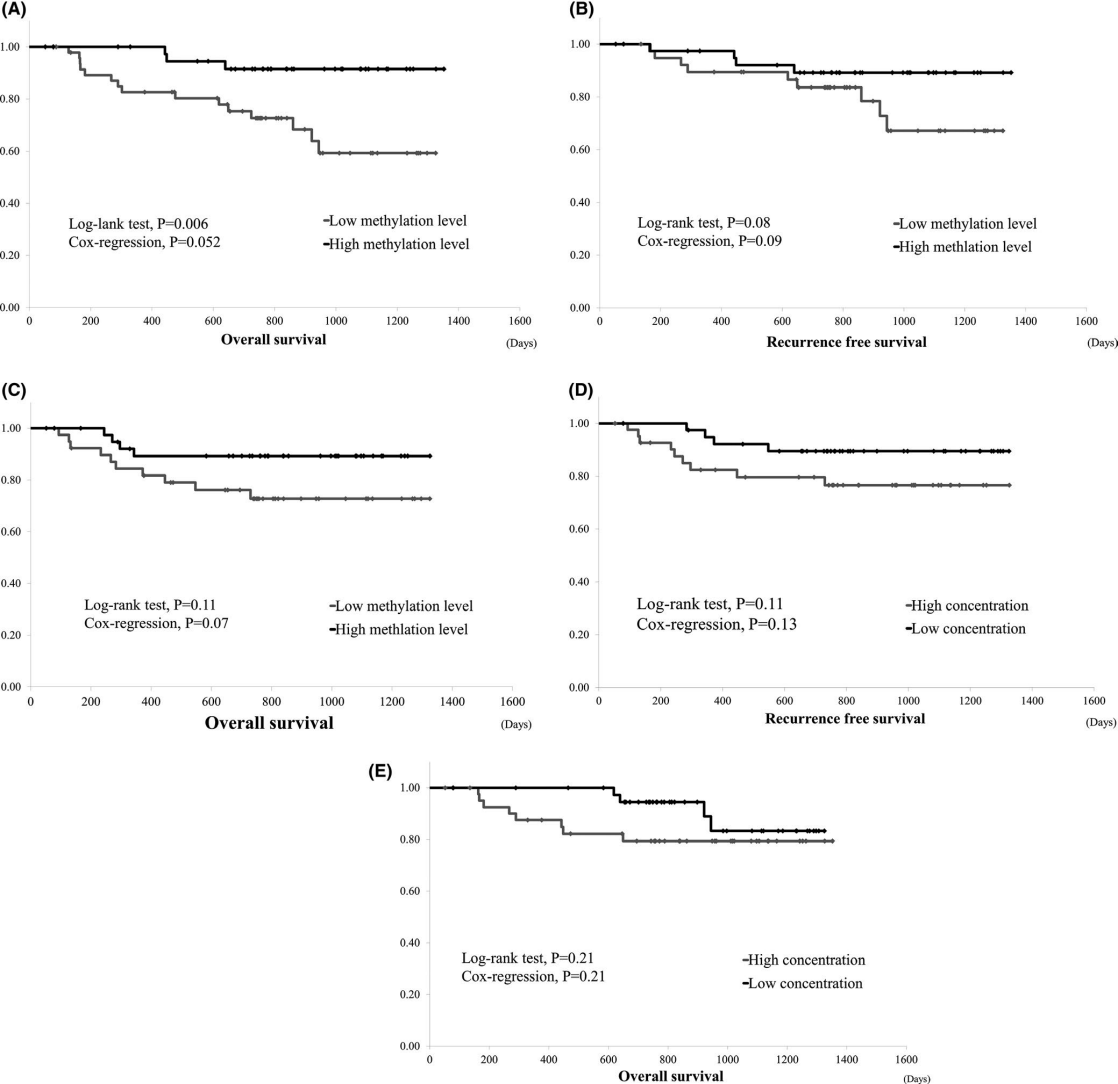
(A) Overall survival of study patients. Overall survival of study patients who did or did not undergo curative surgery. (B) Recurrence‐free survival of patients with curative surgery and methylation levels before surgery. RFS of patients with low methylation levels before surgery tended to be worse than that of patients with high methylation levels, but the difference was not significant. (C) Overall survival of patients with curative surgery and methylation levels before surgery. Overall survival of patients with low methylation levels before surgery tended to be worse than that of patients with high methylation levels, but the difference was not significant. (D) Recurrence‐free survival of patients with curative surgery and concentrations of long LINE‐1 before surgery. Recurrence‐free survival of patients with high concentrations of long LINE‐1 before surgery tended to be worse than that of those with low concentrations of long LINE‐1; however, the difference was not significant. (E) Overall survival of patients with curative surgery and concentrations of long LINE‐1 before surgery. Overall survival of patients with high concentrations of long LINE‐1 before surgery tended to be worse than that of patients with low concentrations of long LINE‐1, but the difference was not significant. RFS, recurrence free survival

The overall survival (OS) of patients with low methylation levels (the median value was set as the cut‐off value) before starting treatment was significantly worse than that of patients with high methylation levels (log‐rank test *p* = 0.006, Cox regression analysis *p* = 0.052; Figure 1A). In 90 patients who underwent curative surgery, 14 (15.6%) patients experienced recurrence, and 13 (14.4%) patients died. Among these 90 patients, the RFS and OS of patients with low methylation levels before surgery were worse than those with high methylation levels; however, there was no significant difference (log‐rank test *p* = 0.08 and *p* = 0.11, Cox regression analysis *p* = 0.09 and *p* = 0.07, respectively; Figure 1B,C). The RFS and OS of patients with high concentrations of long LINE‐1 before surgery were worse than those with low concentrations of long LINE‐1; however, there were no significant differences (log‐rank test *p* = 0.11 and *p* = 0.21, Cox regression analysis *p* = 0.13 and *p* = 0.21, respectively; Figure 1D,E). Multi‐regression analysis showed that only pathological stage is an independent risk factor for RFS and OS of patients receiving curative surgery (Table 3).

**TABLE 3:** Multi‐regression analysis

	RFS		OS	
	*β*	*p*	*β*	*p*
Age	−0.02	0.83	0.02	0.82
Sex	−0.14	0.26	−0.19	0.11
Pathological stage	0.51	<0.001	0.51	<0.001
cfDNA	−0.005	0.96	0.008	0.94
Short LINE‐1 concentration	0.03	0.58	−0.02	0.86
Long LINE‐1 concentration	0.07	0.91	0.05	0.64
cfDNA LINE‐1 methylation level	−0.13	0.26	−0.19	0.07

Abbreviations: OS, overall survival; RFS, recurrence free survival.

### 3.6 Dynamics of long LINE‐1 concentration and LINE‐1 methylation

**FIGURE 2:**
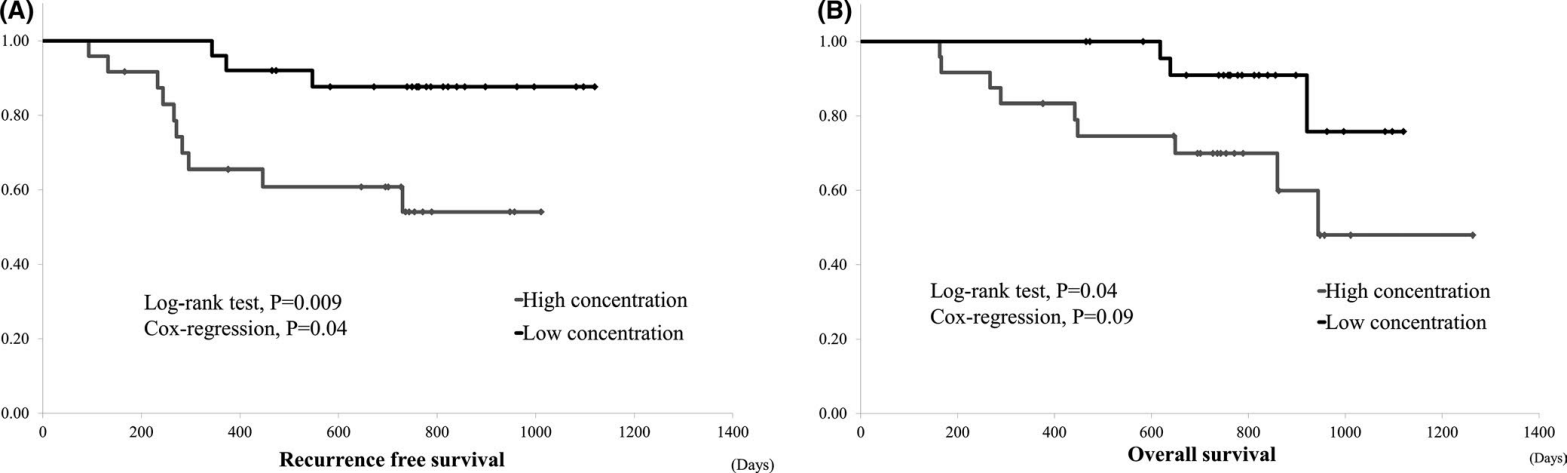
(A) Recurrence‐free survival of patients with curative surgery and concentrations of long LINE‐1 after surgery. Recurrence‐free survival of patients with high concentrations of long LINE‐1 after surgery was significantly worse than that of patients with low concentrations of long LINE‐1. (B) Overall survival of patients with curative surgery and concentrations of long LINE‐1 after surgery. OS of patients with high concentrations of long LINE‐1 after surgery was significantly worse than that of patients with low concentrations of long LINE‐1. OS, overall survival

Postoperative samples were available for 49 patients who underwent curative surgery. Of these 49 patients, 13 (26.5%) experienced recurrence. The RFS and OS of patients with high concentrations of long LINE‐1 (the median value was set as cut‐off value) after surgery were significantly worse than those with low concentrations of long LINE‐1 (log‐rank test *p* = 0.009 and *p* = 0.04, Cox regression analysis *p* = 0.04, *p* = 0.09, respectively; Figure 2A,B). Methylation level after surgery had no impact on PFS (*p* = 0.66) and OS (*p* = 0.43). In the ROC curve for detecting patients who would experience recurrence, the AUCs of postoperative short LINE‐1 concentration, long LINE‐1 concentration, and LINE‐1 methylation were 0.61 (*p* = 0.22), 0.75 (*p* = 0.01), and 0.51 (*p* = 0.95), respectively.

**FIGURE 3:**
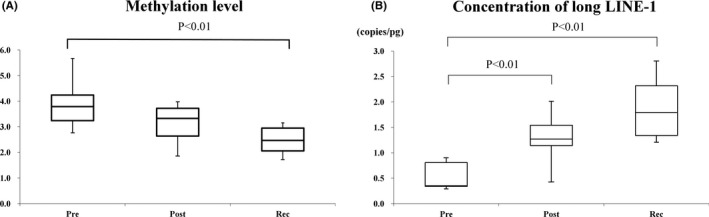
(A) Dynamics of LINE‐1 methylation levels in patients with recurrence. Postsurgery LINE‐1 methylation levels did not differ from those presurgery; however, levels at recurrence were significantly lower than those presurgery. (B) Dynamics of long LINE‐1 concentration in patients with recurrence. Long LINE‐1 concentration postsurgery and at recurrence was significantly higher than that presurgery

Samples at recurrence were available for 12 patients. In these 12, postsurgery LINE‐1 methylation levels did not differ from those presurgery, but levels at recurrence were significantly lower than those presurgery (*p* < 0.01; Figure 3A). Postsurgery long LINE‐1 concentration and that at recurrence were significantly higher than that at presurgery (*p* < 0.01, Figure 3B).

## 4 DISCUSSION

In this study, which evaluated methylation and long LINE‐1 concentrations of cfDNA in patients with gastric cancer, we showed three valuable, novel findings. First, LINE‐1 methylation in cfDNA can be a novel biomarker to screen for gastric cancer. Second, presurgery low LINE‐1 methylation in cfDNA is a negative prognostic biomarker for gastric cancer patients who undergo curative surgery. Finally, methylation levels after surgery do not indicate MRD; conversely, high concentrations of long LINE‐1 postsurgery may indicate.

LINE‐1 methylation in cfDNA can be a novel biomarker for to screen for gastric cancer. LINE‐1 methylation levels in patients with gastric cancer were lower than in patients with benign disease. Remarkably, the methylation levels of patients with stage I gastric cancer were significantly lower than of those with benign disease. It has been reported that LINE‐1 methylation levels in cancerous tissues are lower than those in normal tissue in various carcinomas.^26^, ^27^, ^28^, ^29^ While several studies reported that LINE‐1 methylation levels in the cfDNA of patients with colon^16^ or breast^17^ cancer were lower than in patients without cancer, this study is the first to evaluate patients with gastric cancer. Though not LINE‐1, cfDNA hypomethylation is the distinguishing marker between hepatocellular carcinoma and chronic hepatitis.^30^ Although this study did not meet the sensitivity or specificity thresholds, this less‐invasive method may be promising.

Low LINE‐1 methylation in cfDNA can be a negative prognostic biomarker of gastric cancer patients undergoing curative surgery. In this study, LINE‐1 methylation levels in cfDNA of gastric cancer patients with systemic disease (stage IV) were lower than those of patients with localized disease (stage I–III), and presurgical low LINE‐1 methylation level was a risk factor of recurrence in patients with curative surgery. Patients with low methylation levels showed worse RFS than patients with higher methylation levels, although multiple regression analysis failed to show that low methylation level was an independent risk factor, probably because this study included a small number of patients. In other words, LINE‐1 hypomethylation indicates systemic disease even if patients show no metastasis. Interestingly, tumor size, tumor depth, and lymph node metastasis had no impact on LINE‐1 methylation level in cfDNA. It has been reported that LINE‐1 hypomethylation in cancer tissue is a negative biomarker for prognosis.^15^, ^29^ Similarly, Nagai, et al. reported that LINE‐1 methylation levels in cfDNA are lower in systemic disease compared with localized disease, and the difference in LINE‐1 methylation level between presence of metastasis and tumor size had a significant impact on LINE‐1 methylation level in colorectal cancer.^16^

Methylation levels after surgery do not indicate MRD, but high concentrations of long LINE‐1 after surgery may. Although methylation levels after surgery had no impact on PFS and OS, the RFS and OS of patients with high concentrations of long LINE‐1 after surgery were significantly worse than those with low concentrations of long LINE‐1, consistent with the findings of our previous study in colorectal cancer patients.^2^ As mentioned above, the presence of ctDNA after surgery indicates MRD. However, ctDNA is often not detected in patients with gastric cancer, because most gastric cancer cases show hypo‐mutation. Thus, MRD detection using long LINE‐1 concentration is suitable for patients with gastric cancer. In addition, the cost of detecting long LINE‐1 is much lower than that of ctDNA.

Results from a small number of samples (*n* = 12) suggested that methylation status shows a gradual phased decline. Conversely long LINE‐1 levels gradually increased during the preoperative period to the time of recurrence in cases of recurrence. This phenomenon has not been reported. We speculate that circulating tumor cells may induce this phenomenon. Only serial liquid biopsy can deliver such useful information, and new findings such as this may be critical to identify mechanism of metastasis.

It was reported that cfDNA concentration is remarkably high in patients with metastatic and recurrent colorectal cancer.^23^ However, in this study, long LINE‐1 concentration in patients with gastric cancer was lower than in patients with benign disease, though there were no differences in cfDNA and short LINE‐1 concentrations between patients with gastric cancer and patients with benign disease. Long LINE‐1 concentration may reflect primarily necrosis of cancer cells. Thus, the concentration of long LINE‐1 in patients with benign disease in this study may reflect necrosis of duodenal or gastric ulcers.

The low methylation level of LINE‐1 cfDNA indicates that LINE‐1 of cancer cells is hypomethylated and that the number of those cell is great. Even if the LINE‐1 methylation level of cancer cells is low, and the number of cancer cells is small, the cfDNA LINE‐1 methylation level is not low, because the majority of cfDNA is derived from normal cells. In other words, the low methylation level of LINE‐1 cfDNA indicates that there are numerous, circulating, hypermethylated cancer cells that may have a high potential of growing or metastasizing. The weakness of this assessment method is that LINE‐1 hypomethylation indicates global methylation, but not methylation of specific genes. Thus, by evaluating methylation of specific genes that correlate with prognosis, in addition to evaluation of LINE‐1 methylation, prognostication can be more accurate.

This study had several limitations. First, we only included a small number of patients from a single institution. In particular, we included only eight patients with benign disease. Reliable cut‐off values should be established by a larger study. Second, we did not determine why LINE‐1 hypomethylation in cfDNA indicates systemic disease and why LINE‐1 methylation levels in cfDNA decrease at the time of recurrence. Finally, we did not measure methylation levels in cancer tissue and normal tissue. This was a major limitation of this study.

In conclusion, presurgical hypomethylation of LINE‐1 in cfDNA is a negative prognostic factor and the methylation level decreases further at the time of recurrence. High concentrations of long LINE‐1 after curative surgery indicate MRD and high risk of recurrence. Long LINE‐1 concentration increases at the time of recurrence. Serial liquid biopsy to detect LINE‐1 methylation levels and long LINE‐1 concentration can yield useful information for determining the treatment strategy for patients with gastric cancer. These approaches represent an attractive biomarker for hypo‐mutated cancer in which ctDNA is detected at low levels.

## CONFLICT OF INTEREST

The authors have no conflicts of interest.

## AUTHOR CONTRIBUTIONS

Kazuhide Ko: Conceptualization, formal analysis, investigation, methodology writing—original draft. Yoshikazu Kananazawa: Conceptualization, Takeshi Yamada: Conceptualization, formal analysis, writing—original draft. Daisuke Kakinuma: formal analysis and investigation. Kunihiko Matsuno Conceptualization, formal analysis, investigation, and methodology. Fumihiko Ando: Formal analysis and investigation. Sho Kuriyama: Writing—original draft, Akihisa Matsuda: Writing—review and editing. Hiroshi Yoshida: Supervision.

## INFORMED CONSENT

This study was carried out in accordance with the Declaration of Helsinki. The study protocol was approved by the ethics review committee of Nippon Medical School (Tokyo, Japan, approval number: 28‐03‐738). Written informed consent was obtained from each patient.

## Data Availability

Datasets used and/or analyzed during this study are available from the corresponding author.
